# Photoautotrophic cultivation of a *Chlamydomonas reinhardtii* mutant with zeaxanthin as the sole xanthophyll

**DOI:** 10.1186/s13068-024-02483-8

**Published:** 2024-03-14

**Authors:** Minjae Kim, Stefano Cazzaniga, Junhwan Jang, Matteo Pivato, Gueeda Kim, Matteo Ballottari, EonSeon Jin

**Affiliations:** 1 Department of Life Science, Research Institute for Natural Sciences, Hanyang University, Seoul, 04763 Korea; 2 Dipartimento di Biotecnologie, Università di Verona, Verona, Italy; 3 Hanyang Institute of Bioscience and Biotechnology, Hanyang University, Seoul, 04763 Korea

**Keywords:** Microalgae, Photoautotrophic cultivation, Self-shading effect, Xanthophyll mutant, antenna truncation

## Abstract

**Background:**

Photosynthetic microalgae are known for their sustainable and eco-friendly potential to convert carbon dioxide into valuable products. Nevertheless, the challenge of self-shading due to high cell density has been identified as a drawback, hampering productivity in sustainable photoautotrophic mass cultivation. To address this issue, mutants with altered pigment composition have been proposed to allow a more efficient light diffusion but further study on the role of the different pigments is still needed to correctly engineer this process.

**Results:**

We here investigated the *Chlamydomonas reinhardtii Δzl* mutant with zeaxanthin as the sole xanthophyll. The *Δzl* mutant displayed altered pigment composition, characterized by lower chlorophyll content, higher chlorophyll a/b ratio, and lower chlorophyll/carotenoid ratio compared to the wild type (Wt). The *Δzl* mutant also exhibited a significant decrease in the light-harvesting complex II/Photosystem II ratio (LHCII/PSII) and the absence of trimeric LHCIIs. This significantly affects the organization and stability of PSII supercomplexes. Consequently, the estimated functional antenna size of PSII in the *Δzl* mutant was approximately 60% smaller compared to that of Wt, and reduced PSII activity was evident in this mutant. Notably, the *Δzl* mutant showed impaired non-photochemical quenching. However, the *Δzl* mutant compensated by exhibiting enhanced cyclic electron flow compared to Wt, seemingly offsetting the impaired PSII functionality. Consequently, the *Δzl* mutant achieved significantly higher cell densities than Wt under high-light conditions.

**Conclusions:**

Our findings highlight significant changes in pigment content and pigment–protein complexes in the *Δzl* mutant compared to Wt, resulting in an advantage for high-density photoautotrophic cultivation. This advantage is attributed to the decreased chlorophyll content of the *Δzl* mutant, allowing better light penetration. In addition, the accumulated zeaxanthin in the mutant could serve as an antioxidant, offering protection against reactive oxygen species generated by chlorophylls.

**Supplementary Information:**

The online version contains supplementary material available at 10.1186/s13068-024-02483-8.

## Introduction

Microalgae are sustainable and eco-friendly resources that convert CO_2_ into useful compounds, such as biofuels and carotenoids [1]. Large-scale cultivation has less growth efficiency owing to a self-shading or mutual shading effect that inevitably occurs during high-density cultivation [2, 3]. An increase in light intensity cannot overcome this issue, because (i) the excess light energy absorbed by the external layer is wasted as fluorescence or heat emission [4] and (ii) excess light increases the risk of photodamage and photoinhibition [5].

Decreasing algal optical density to facilitate light diffusion into the inner layers has been suggested as a possible solution to improve light penetration and biomass productivity [6]. This can be achieved with “paler” strains with less pigment content [7–11]. These strains absorb only a small fraction of the available light, allowing the remaining light to diffuse to other layers of the photobioreactor [8]. Among the different pigments present in the photosynthetic membranes, the pigments bound to the external antenna complexes are those that can be safely decreased, while the pigments bound to the core complexes of the photosystems are crucial for photosynthesis, being the site of the photochemical reactions. Accordingly, the mutants with truncated Photosystem II (PSII) antenna grow faster and reach higher cellular concentrations than their parental strains [8, 9].

Another factor that negatively affects productivity is photoinhibition, owing to excess light energy absorbed by the photosystems, which results in photodamage. Photoinhibition is an intrinsic effect of microalgae growing inside a photobioreactor, where the bubbling and mixing system exposes the algae to sudden changes in light intensity, resulting in temporary saturation of the electron transport chain and generation of reactive oxygen species (ROS) that damage cell macromolecules, limiting productivity [12]. As a solution to photoinhibition, increasing the resistance to excess absorbed light energy and ROS generation could be helpful. A viable strategy to generate algal lines with these characteristics is to modify the carotenoid composition [13, 14].

Carotenoids are divided into carotenes and xanthophylls and are distributed in different locations of the photosynthetic apparatus [15]: β-carotene is bound by the protein of the core complex and the antenna of Photosystem I (PSI), whereas xanthophylls are bound only in the light-harvesting complex (LHC). In *Chlamydomonas reinhardtii*, different xanthophylls are present [16]: the β–β xanthophylls zeaxanthin, antheraxanthin, violaxanthin, and neoxanthin and the ε–β xanthophylls loroxanthin and lutein. All the β–β xanthophylls are derived from β-carotene that is generated from the action of lycopene β-cyclase (LCYB) on lycopene, whereas the ε–β xanthophylls are obtained by the combined action of LCYB and lycopene ε-cyclase (LCYE) [17]. Under normal light, zeaxanthin does not steadily accumulate, but is immediately converted into violaxanthin by zeaxanthin epoxidase (ZEP) through transient mono-epoxidated antheraxanthin [17]. When the light intensity is increased, a portion of violaxanthin is converted back into zeaxanthin. In the photosynthetic apparatus, the two photosystems are organized into a reaction center and the LHC, which serve as antennae that increase the rate of light energy capture and efficiently transfer it to the reaction center [18]. The protein complexes of the photosystems bind chlorophyll a (the main pigment responsible for light absorption) and b [19]. Carotenoids play multiple roles in photosynthesis as additional pigments in photosynthetic complexes [20]. Carotenoids increase light harvesting and maintain the structure and function of photosystems [19]. They also contribute to photoprotection by scavenging ROS and dissipating energy absorbed in excess through a mechanism called non-photochemical quenching (NPQ) [4].

In *C. reinhardtii*, a spontaneous mutant named *npq2lor1* contains only β-carotene and zeaxanthin as carotenoids [16]. The constitutive production of zeaxanthin, rather than triggered by the light regime, as in the case of the wild type (Wt), is an interesting feature of the *npq2lor1* mutant, because zeaxanthin has a high nutritional value, as required by human eye health [21]. Importantly, the change in carotenoids in the *npq2lor1* mutant was reported to not significantly compromise photon conversion efficiency, but resulting in a truncated light-harvesting antenna size for PSII [16]. However, mutant strains generated by random mutagenesis have been reported to carry multiple mutations, which could lead to a complex genotype to phenotype correlation [22, 23]. Therefore, the use of target-specific mutations can effectively address these concerns.

The CRISPR–Cas9 method has gained prominence in *C. reinhardtii* for its utility in achieving target-specific mutagenesis [24–26]. Recently, we employed this method to create a double knockout mutant of the *LCYE* and *ZEP* genes in *C. reinhardtii* and named it as *Δzl* [27]. Our objective was to gain deeper insights into the role of carotenoids in *C. reinhardtii*, in comparison to the *npq2lor1* mutant, which was obtained through random insertional mutagenesis [16]. In this study, we investigated the growth pattern and photosynthetic characteristics of the *Δzl* mutant under photoautotrophic conditions. The *Δzl* mutant exhibited a higher maximum cell density compared to the Wt strain under high-light conditions, indicating that the *Δzl* mutant resulting from having only zeaxanthin as xanthophylls may be advantageous for photoautotrophic large-scale cultivation.

## Results

### *Pigment content and pigment–protein complex stoichiometry of Wt and the *Δzl* mutant*

The change in xanthophyll composition in the mutant affected chlorophyll and carotenoid distribution (Table 1). The *Δzl* mutant displayed ~ 60% lower chlorophyll content per cell (1.75 vs 0.67 in Wt and *Δzl*, respectively), higher chlorophyll a/b ratio (Chl a/b; 2.82 vs 3.65 in Wt and *Δzl*, respectively), and lower chlorophyll/carotenoid ratio (Chl/Car; 2.91 vs 2.28 in Wt and *Δzl*, respectively) compared to Wt.

**Table 1:** Pigment contents of Wt and the *Δzl* mutant grown in HS medium at 100 μmol photons m^−2^ s^−1^

	Wt	*Δzl*
Total chlorophyll (pg cell^−1^)	1.75 ± 0.14	0.67 ± 0.04*
Total carotenoid (pg cell^−1^)	0.40 ± 0.04	0.20 ± 0.11*
Chlorophyll a/b	2.82 ± 0.03	3.65 ± 0.16*
Chlorophyll/Carotenoid	2.91 ± 0.12	2.28 ± 0.02*

All the experiment was performed in biological replicates (*n* = 3). Statistical analysis was performed using Student’s *t* test (*p < 0.05)

**Fig. 1:**
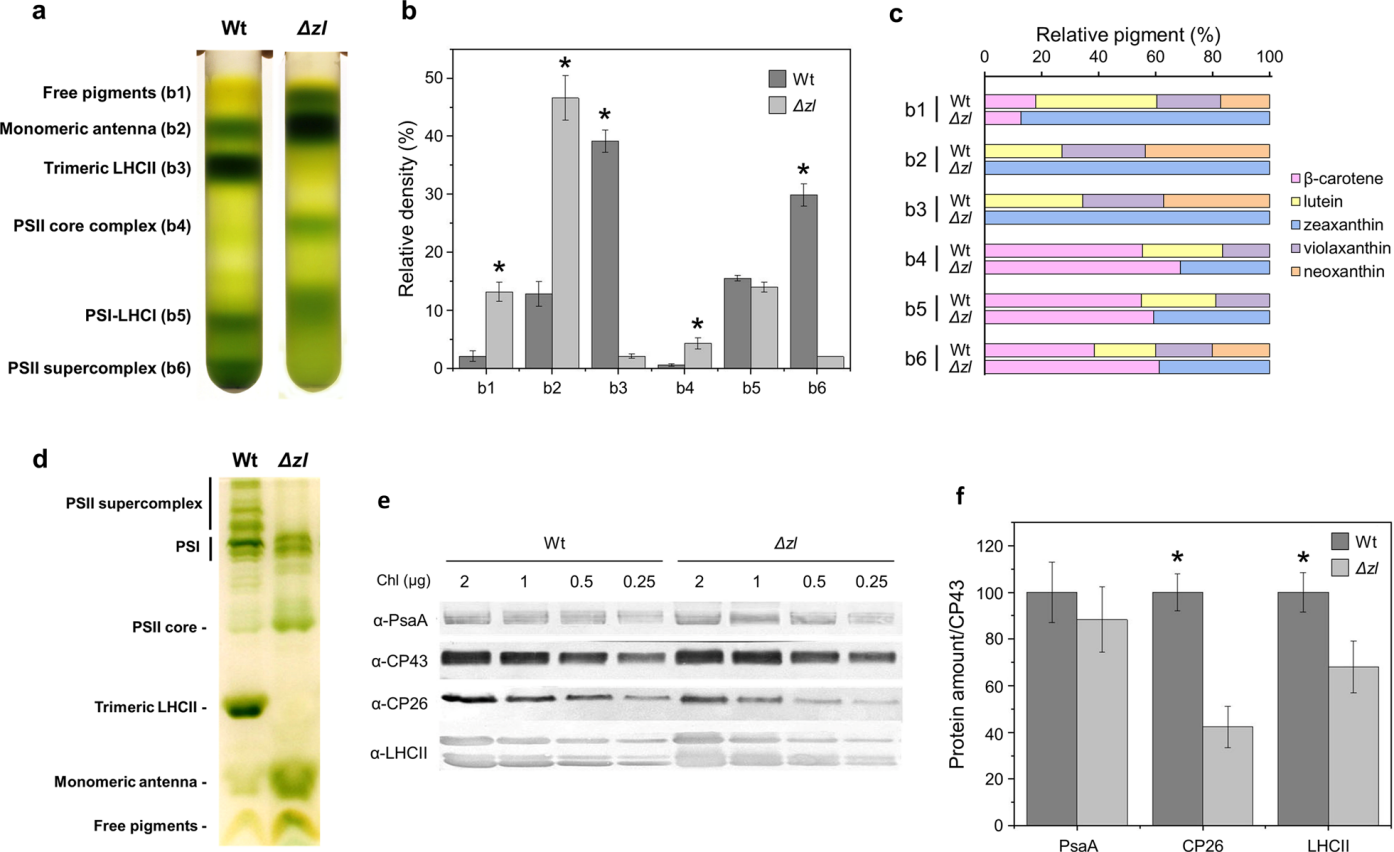
Organization of photosynthetic complexes of Wt and the *Δzl* mutant. **a** Sucrose density gradient fractionation of Wt and *Δzl* solubilized with 0.6% dodecyl‐α‐D‐maltoside (α‐DM). The composition of the green bands is indicated on the left. **b** Distribution as percentages of the chlorophylls in the gradient fractions. **c** Carotenoid distribution in the different gradient fractions. **d** Thylakoid pigment–protein complexes separated by non-denaturing Deriphat PAGE. **e** Western blotting images used for immunotitration of thylakoid proteins using antibodies against PsaA, CP43, CP26, and LHCII. Chlorophylls (2, 1, 0.5, and 0.25 µg) were loaded on cellulose membrane. **f** Immunotitration of thylakoid proteins. Data were corrected by CP43 and normalized to the Wt ratio. All the experiment was performed in biological replicates (*n* = 3)

The effect of this change in pigment content on the organization of photosynthetic complexes was investigated. Thylakoid membranes were isolated from the Wt and the *Δzl* mutant and solubilized. Different chlorophyll-binding complexes were separated by ultracentrifugation on a sucrose gradient (Fig. 1a, b). The obtained fractions corresponded, from top to bottom of sucrose gradients, to free pigments (b1), monomeric LHC (b2), trimeric LHCII (b3), PSII core complex (b4), PSI–LHCI (b5), and PSII supercomplexes (b6). The absorption spectra of each fraction from both genotypes are shown in Additional file 1: Fig. S1. The carotenoid distribution in each band was determined using HPLC (Fig. 1c). In the Wt, three different xanthophylls were detected in the fraction corresponding to the antenna protein (b2–3): neoxanthin, violaxanthin, and lutein. Zeaxanthin was absent in the Wt, because it did not accumulate under low-light conditions. Differently, in the *Δzl* mutant, zeaxanthin was the only carotenoid present. The absence of b3 from the *Δzl* mutant corroborates the pivotal role of lutein in trimer stability. Therefore, b2, which contains the antenna in the monomeric state, is highly increased, because it contains all dissociated LHCII. Changes in carotenoids and the absence of trimeric LHCII also affected the stability of the PSII supercomplexes (b6), which were almost absent from the *Δzl* mutant, whereas the intensity of the band containing the dissociated PSII core (b4) was highly increased (Fig. 1b). Also, the increase in the fraction corresponding to free pigments (b1) in the mutant suggests that the folding of LHC complexes in the presence of zeaxanthin as the sole xanthophyll is somewhat less efficient. For the PSI–LHC1 complex (b5), there was no significant difference in the native absorption spectra as well as the ratio of β-carotene mediating the binding by the core and antenna subunits. It suggests a similar PSI assembly in both strains.

The absence of PSII supercomplexes was confirmed also by non-denaturing Deriphat–polyacrylamide gel electrophoresis (Deriphat–PAGE), after solubilization of thylakoid membranes using dodecyl-α-D-maltoside (α-DM) (Fig. 1d). The non-denaturing electrophoresis allowed the separation of PSII supercomplexes into different bands corresponding to the dimeric core associated with different states of antenna protein aggregation. Four PSII bands were present in the Wt, but they were completely absent in the *Δzl* mutant, suggesting partial destabilization of PSII supercomplexes. Deriphat–PAGE confirmed the absence of trimeric LHCII and an increase in the intensity of the band corresponding to the PSII core.

The stoichiometry of the thylakoid proteins was validated using specific antibodies against PsaA, LHCII, CP26, and CP43 (Fig. 1e). PsaA and CP43 were used as representative proteins of PSI and PSII, respectively. The LHCII content was investigated using an antibody recognizing different LHCBM subunits of *C. reinhardtii* [28]. In the *Δzl* mutant, the PSI/PSII ratio was similar to the Wt, whereas the LHCII/PSII content decreased significantly (Fig. 1f). As for LHCII, the monomeric subunit CP26 was strongly decreased in the *Δzl* mutant with a CP26/CP43 ratio that was ~ 40% that of the Wt. These results indicate also that the reduction in chlorophyll content per cell in the *Δzl* mutant causes a proportional decrease of PSI and PSII per cell. Consequently, these data confirmed the antenna truncation in the mutant with zeaxanthin as the sole xanthophyll.

### Light-harvesting efficiency and photosynthetic activity of Wt and the Δzl mutant

Light-harvesting efficiency of Wt and the *Δzl* mutant was evaluated by measuring the chlorophyll fluorescence in limiting light upon 3-(3,4-dichlorophenyl)-1,1-dimethylurea (DCMU) treatment. DCMU is an inhibitor of the PSII electron transport and the kinetics of chlorophyll fluorescence emission in DCMU-treated cells are inversely proportional to the light-harvesting capacity of PSII. This capacity is the PSII functional antenna size and is calculated as the reciprocal of the time required to reach two-thirds of the maximal fluorescence emission [29]. According to this calculation, the *Δzl* mutant showed ~ 40% lower light harvesting capacity, compared to the Wt (Additional file 2: Fig. S2) in accordance with the decreased LHCII and CP26 contents.

**Fig. 2:**
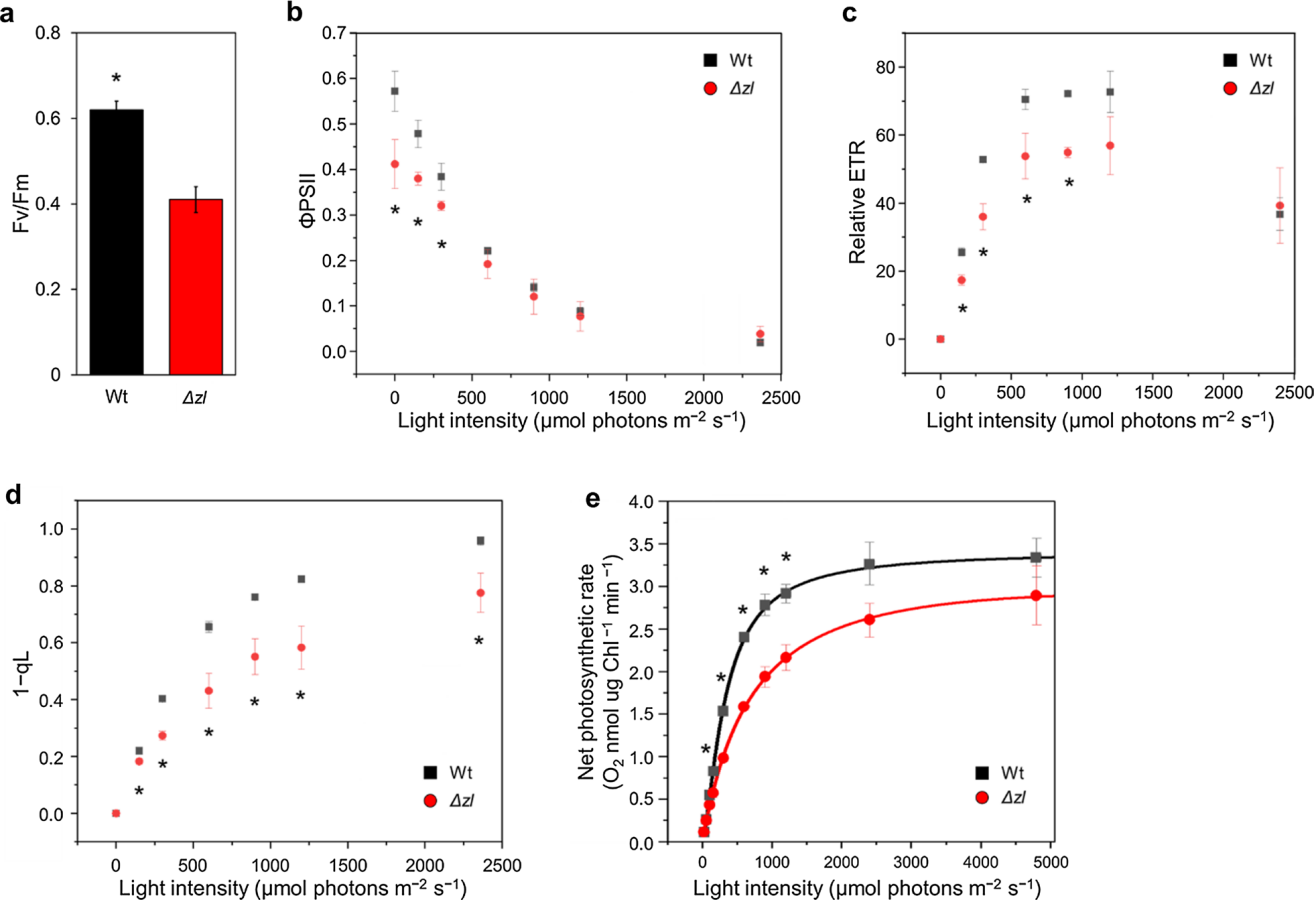
Photosynthetic parameters. **a** Maximum quantum efficiency of photosystem II photochemistry (Fv/Fm). **b** PSII operating efficiency (ΦPSII). **c** Relative electron transport rate (ETR). **d** Photochemical quenching (1‐qL) and (**e**) photosynthetic oxygen evolution at different actinic light intensities. Net photosynthetic rate data were fitted with the Hill equation. All data were collected from the cells grown in HS medium at 100 μmol photons m^−2^ s^−1^. All the experiment was performed in biological replicates (*n* > 3). Statistical analysis was performed using Student’s *t* test (*p < 0.05)

Photosynthetic activities of the Wt and the *Δzl* mutant were compared at different light intensities while monitoring PSII operating efficiency (ΦPSII), electron transport rate (ETR), and photochemical quenching (1−qL) [30]. The Fv/Fm in the *Δzl* mutant was considerably lower than in the Wt (Fig. 2a). Consistently, the ΦPSII and ETR in the *Δzl* mutant were significantly lower than those of Wt but became not significantly different at light intensities higher than 900 μmol photons m^−2^ s^−1^ (Fig. 2b, c). In addition, a strong decrease in photochemical quenching was observed in the *Δzl* mutant (Fig. 2d). Photosynthetic activity was investigated under different actinic lights to monitor oxygen evolution. The oxygen production rate of the *Δzl* mutant was lower than that of the Wt at most light intensities, except for those below 50 μmol photons m^−2^ s^−1^ (Fig. 2e). The maximum value of oxygen production (Pmax) was lower (3.34 in the Wt vs. 2.90 in the *Δzl* mutant) and the light intensity needed to reach half the saturation of the oxygen evolution was higher in the mutant than in the Wt. The slope of linear increase, in the phase when oxygen evolution is proportional to the light intensity, was lower in the *Δzl* mutant (Additional file 7: Table S1). These results indicated that the overall photosynthetic parameter decreased in the *Δzl* mutant owing to a smaller antenna size.

### Cyclic electron flow around the PSI of Wt and the Δzl mutant

**Fig. 3:**
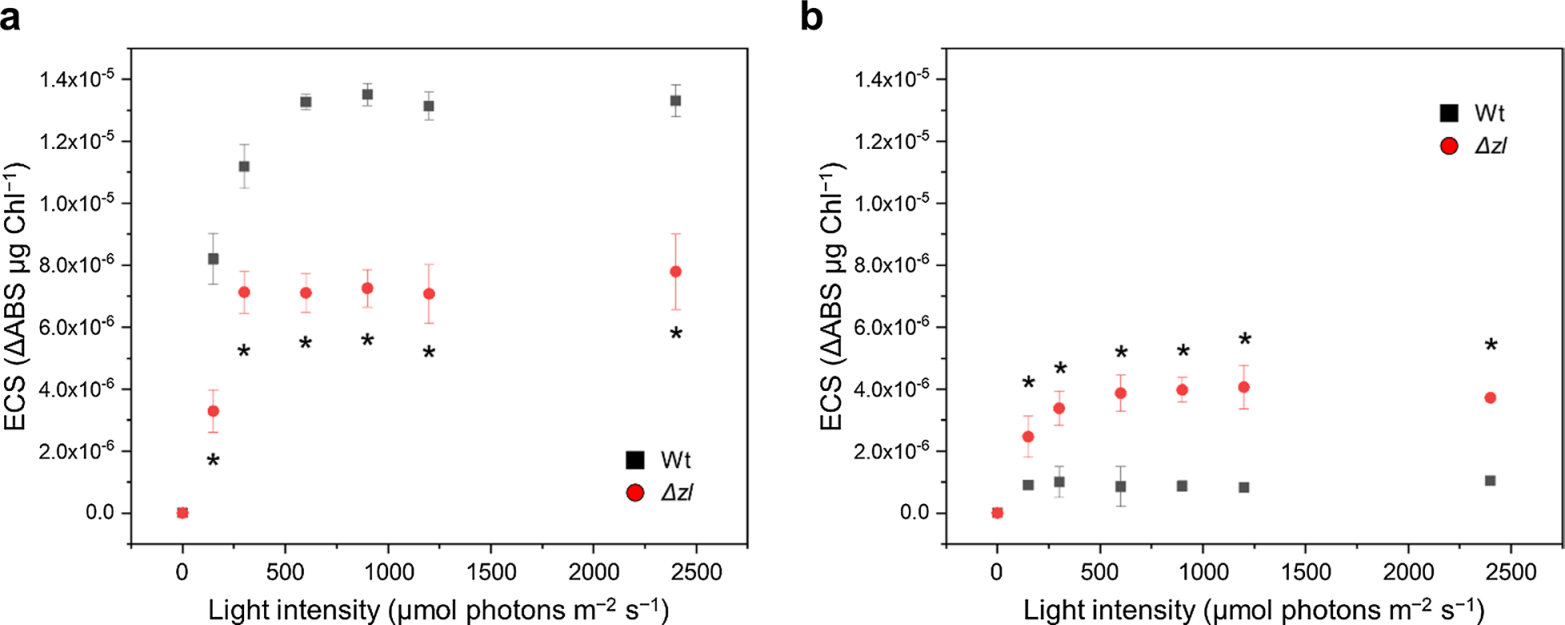
Total proton motive force of Wt and the *Δzl* mutant. Electrochromic shift (ECS) of the carotenoid absorption spectrum was measured upon exposure to different light intensities, without (**a**) or with (**b**) the addition of DCMU. All the experiment was performed in biological replicates (*n* = 3). Statistical analysis was performed using Student’s *t* test (*p < 0.05)

Photosynthetic electron transport is coupled with the generation of a proton gradient across the thylakoid membrane, which is exploited as a proton motive force (PMF) to produce ATP by ATPases [31]. The PMF was measured under different actinic lights and monitored for a light-dependent carotenoid absorption electrochromic shift (ECS) [32, 33]. In the *Δzl* mutant, there was a lower ECS under all the light conditions tested in accordance with a lower ETR. The ECS was also measured in the presence of DCMU to inhibit PSII and the linear electron flow from PSII to PSI. The residual PMF is related to the cyclic electron flow (CEF) around the PSI. In the Wt, the ECS signal decreased strongly after DCMU treatment, whereas in the mutant, approximately 50% of the signal was still present and maintained a value considerably higher than in the Wt (Fig. 3), suggesting an increased CEF in the *Δzl* mutant.

### Non-photochemical quenching of Wt and the Δzl mutant

**Fig. 4:**
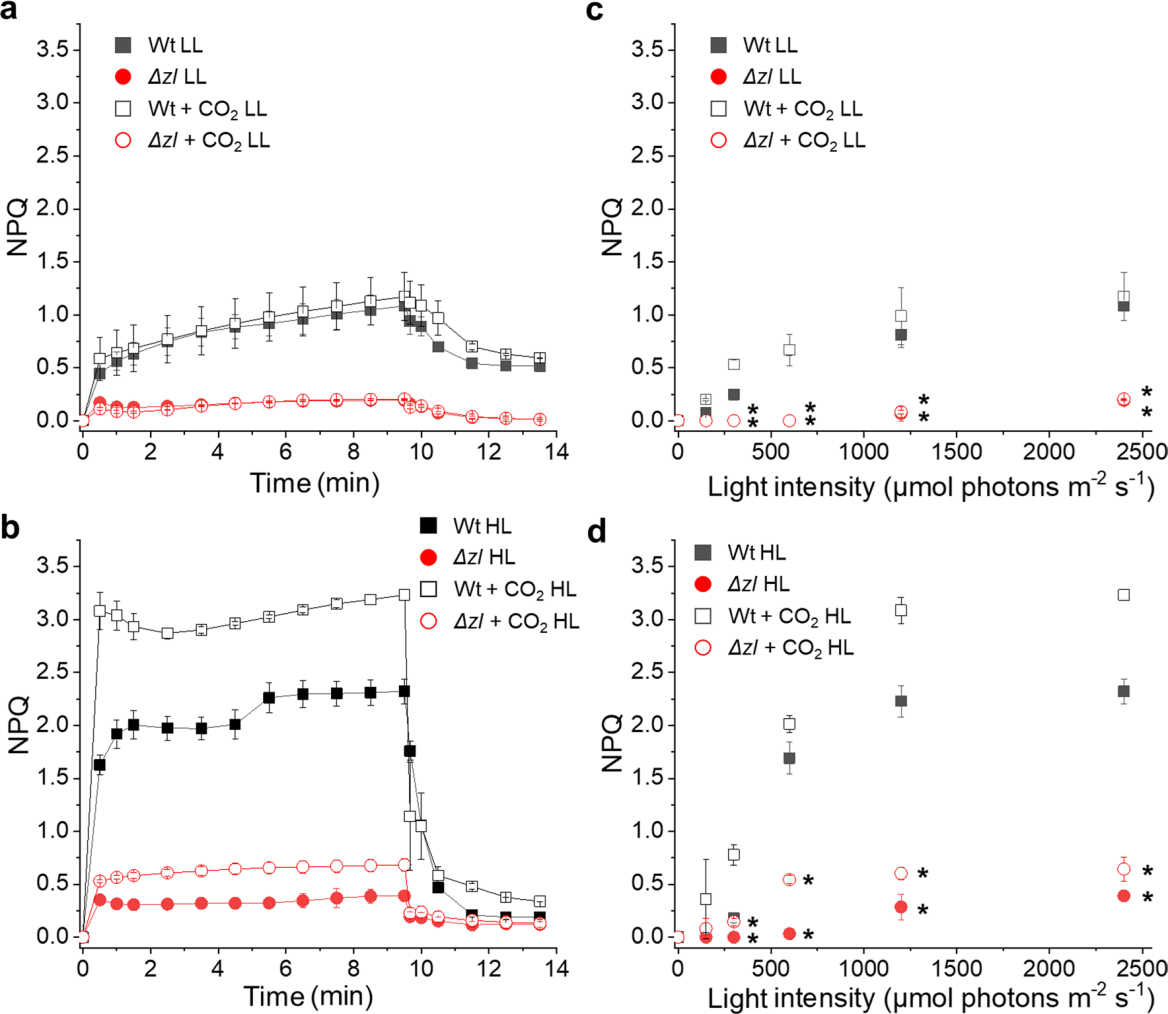
Non-photochemical quenching (NPQ) of Wt and the *Δzl* mutant. **a**, **b** Measurement of NPQ kinetics of low light (LL, **a**) or high light (HL, **b**) acclimated cells using actinic lights of 2400 μmol photons m^−2^ s^−1^. **c**, **d** NPQ values after 10 min of illumination with different actinic light intensities measured in low light (LL, **c**) or high light (HL, **d**) acclimated cells. Closed symbol refers to cells grown at atmospheric CO_2_, while open symbols to cells grown at 5% CO_2_. All the experiment was performed in biological replicates (*n* = 4). Statistical analysis was performed using Student’s *t* test (*p < 0.05)

The consequence of the altered xanthophyll distribution was then evaluated in terms of photoprotection by measuring NPQ in the Wt and the *Δzl* mutant. NPQ was monitored in low light (50 µmol photons m^−2^ s^−1^) or high light (1200 µmol photons m^−2^ s^−1^) adapted cells grown in closed photobioreactors bubbled with ambient air (~ 0.04% CO_2_ concentration) or with air enriched in CO_2_ (5% final CO_2_ concentration), because CO_2_ availability could have an impact on the quenching. (Fig. 4a, b). In LL acclimated cells, a maximum NPQ value of 1 was measured in the Wt upon exposure to actinic light of 2400 µmol photons m^−2^ s^−1^. The additional CO_2_ did not have a major effect on the NPQ curve at the higher actinic light, while at the lower light intensities (below 300 µmol photons m^−2^ s^−1^) it increased the quenching. At all the actinic light tested the mutant showed a strongly decreased NPQ compared to the Wt case (Fig. 4a, b; Additional file 3: Fig. S3). In HL samples the Wt reached a higher NPQ compared to LL, as expected, because HL acclimation induces the expression of *LHCSR*, a trigger for NPQ in *C. reinhardtii*. Even in the case of HL cells, *∆zl* mutant exhibited a strongly decreased NPQ compared to Wt at all the actinic light used (Fig. 4c, d; Additional file 4: Fig. S4). In HL acclimated cells the addition of CO_2_ slightly increased the quenching in both Wt and the mutant, but even in this case the NPQ measured for *∆zl* mutant was always lower compared to the Wt case. These data show that the NPQ mechanism was strongly impaired in the *Δzl* mutant.

### Growth pattern of Wt and the Δzl mutant in photoautotrophic conditions

**Fig. 5:**
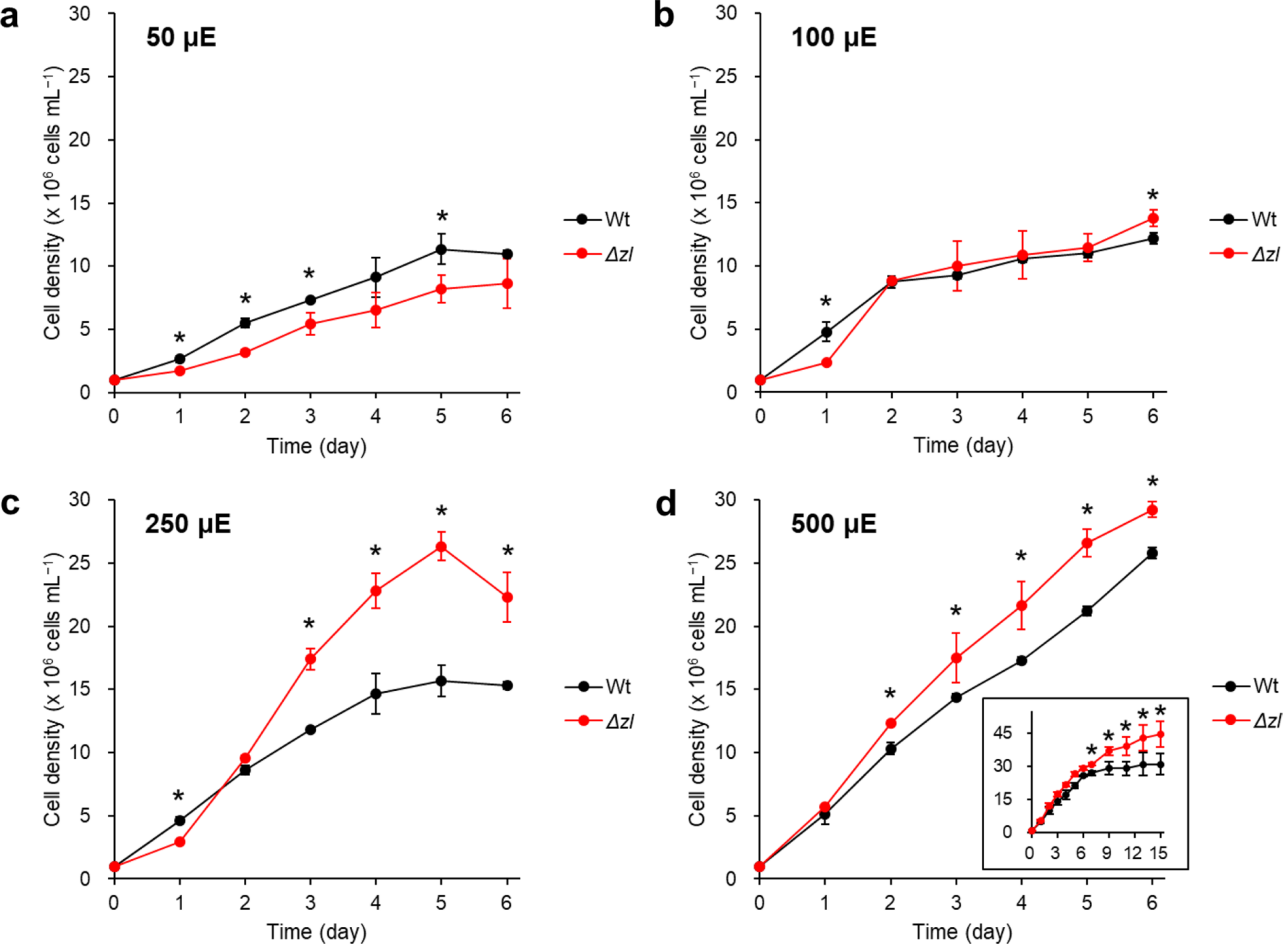
Growth patterns of Wt and the *Δzl* mutant in photoautotrophic cultivation (ambient air). The carbon source was supplied by naturally dissolved air during agitating. Cell growth was measured under 50 ± 5 µmol photons m^−2^ s^−1^ (**a**), 100 ± 10 µmol photons m^−2^ s^−1^ (**b**), 250 ± 30 µmol photons m^−2^ s^−1^ (**c**), and 500 ± 50 µmol photons m^−2^ s^−1^ (**d**). The long-term experiment at 500 ± 50 µmol photons m^−2^ s^−1^ was displayed in the box. All the experiment was performed in biological replicates (*n* > 2). Statistical analysis was performed using Student’s *t* test (*p < 0.05)

We compared the growth of Wt and the *Δzl* mutant in relation to inorganic carbon availability (ambient air (~ 0.04%) and 5% CO_2_). Initially, cells were cultured under conditions where only CO_2_ in ambient air was available being naturally dissolved by flask agitation (Fig. 5). To determine the effect of light intensity, we monitored growth over 6 days at four different light intensities: 50 ± 5, 100 ± 10, 250 ± 30, and 500 ± 50 µmol photons m^−2^ s^−1^. Except at 50 μmol photons m^−2^ s^−1^, *Δzl* mutant exhibited an increase in maximum cell density at all other tested light intensities compared to Wt. Notably, at 100 ± 10 µmol photons m^−2^ s^−1^, the maximum cell density of *Δzl* surpassed that of Wt showing a significant rise in cell density. At 250 µmol photons m^−2^ s^−1^, the *Δzl* mutant achieved a cell density over 45% higher than Wt. This difference in growth was even more evident at the highest light intensity of 500 ± 50 µmol photons m^−2^ s^−1^. The extended growth period shown in Fig. 5d clearly illustrates that the *Δzl* mutant maintains robust growth beyond the stationary phase typically observed in Wt.

**Fig. 6:**
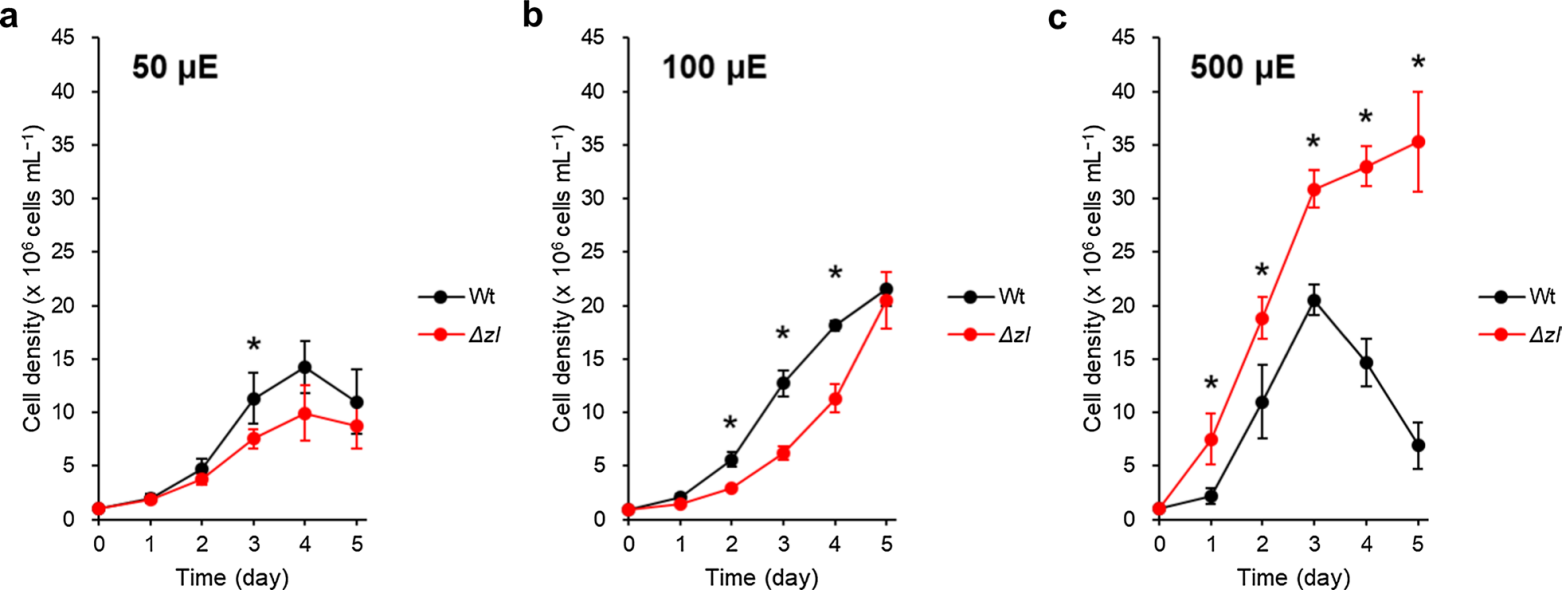
Growth patterns of Wt and the *Δzl* mutant in photoautotrophic cultivation (5% CO_2_). The carbon source was supplied by 5% CO_2_ bubbling. Cell growth was measured under 50 ± 5 µmol photons m^−2^ s^−1^ (**a**), 100 ± 10 µmol photons m^−2^ s^−1^ (**b**), and 500 ± 50 µmol photons m^−2^ s^−1^ (**c**). All the experiment was performed in biological replicates (*n* > 2). Statistical analysis was performed using Student’s *t* test (*p < 0.05)

Next, we examined cell growth at higher CO_2_ availability providing air with CO_2_ concentration increased at 5%. We chose to experiment with three specific light intensity ranges: 50 ± 5, 100 ± 10, and 500 ± 50 µmol photons m^−2^ s^−1^. The results from these tests are presented in Fig. 6. With high CO_2_ availability, cell growth rates of both strains tended to be faster, but at 50 and 100 µmol photons m^−2^ s^−1^, the *Δzl* mutant grew more slowly than the Wt. Interestingly, at 500 µmol photons m^−2^ s^−1^, the Wt cells peaked in density on day 3, whereas the cell density of the *Δzl* mutant increased even after day 3.

Taken together, these results indicate that the *Δzl* mutant sustains a growth advantage, consistently reaching greater cell densities in the high light conditions, regardless of CO_2_ concentrations.

## Discussion

Herein, we investigated the *C. reinhardtii Δzl* mutant obtained from a target-specific double knockout of the ZEP and LCYE genes. Previously, the *npq2lor1* mutant was obtained by mutagenesis of the *lor1* mutant by phenotypic screening [16]. However, random mutagenesis followed by phenotypic screening may generate hundreds of mutations resulting in unpredictable gene mutations and growth conditions [22, 23]. Therefore, the characterization of the *Δzl* mutant helps to clarify and update the previous knowledge obtained from the *npq2lor1* mutant. In addition, we discuss the advantages of the *Δzl* mutant, which has a smaller antenna for photoautotrophic cultivation based on the photosynthetic parameters analysis.

### Δzl mutation affected the organization of the PSII supercomplexes

The LHCII/PSII and CP26/PSII contents were strongly diminished in the *Δzl* mutant and trimeric LHCIIs were completely destabilized. LHCII binds 14 chlorophyll molecules and four molecules of carotenoids (two lutein molecules, one violaxanthin molecule, and one neoxanthin molecule) [35]. The carotenoid-binding sites are specific for different xanthophylls; L1 and L2 at the center of the protein bind lutein, whereas the external sites V1 and N1 bind violaxanthin (or zeaxanthin) and neoxanthin, respectively [36]. Studies on different mutants and in vitro reconstitution of antennae proteins have shown that lutein, violaxanthin, and zeaxanthin can bind to sites L1 and L2, but trimers can only be formed if lutein is present [37]. This indicates that LHCII trimer formation in vivo requires lutein alone, which is sufficient for trimerization. Indeed, the LHCII proteins seem to be present in the *Δzl* mutant but essentially in a monomeric state. Consequently, the absence of an LHCII trimer in the *Δzl* mutant affected the organization of the PSII supercomplexes and their stability.

### Δzl mutation weakened the chlorophyll stability of the PSII antenna

The decreased LHCII/PSII ratio in the *Δzl* mutant affected its PSII light-harvesting capacity. The estimated functional antenna size of PSII in the *Δzl* mutant was approximately 60% that of the Wt (Additional file 2: Fig. S2). The PSI/PSII ratio remained comparable between the two genotypes, suggesting that the decrease in chlorophyll had a uniform impact on both photosystems in the *Δzl* mutant.

The chlorophyll content was markedly lower in the *Δzl* mutant (0.67 pg/cell) than Wt (1.75 pg/cell), likely related to the altered carotenoid composition and diminished PSII supercomplexes. Moreover, the Chl a/b ratio increased because of a decrease in chlorophyll b-binding antenna subunits. The Chl/Car ratio was decreased in the *Δzl* mutant, implying a higher decrease of chlorophylls with respect to carotenoids. Carotenoids can also be present in the membrane, where they function as scavengers of ROS, whereas chlorophyll is maintained only inside the subunits of photosynthetic complexes [4, 20, 36, 37]. Nevertheless, not only chlorophyll but also carotenoid content was decreased on a cell basis in the *Δzl* mutant, even if the effect was stronger on the chlorophyll content per cell.

### Δzl mutation caused a proportional decrease in PSI and PSII

The *Δzl* mutant showed a decreased chlorophyll content (60% less than Wt) but the ratio between PSII and PSI, measured by immunotitration or native complexes densitometry, was similar between the two genotypes. These results indicate that the reduction in chlorophyll content per cell in the *Δzl* mutant causes a proportional decrease in PSI and PSII per cell. To evaluate if the change of xanthophyll composition has an effect also on the functionality of PSI, its photochemical activity was measured on complexes isolated on the sucrose gradient following the kinetics of P700 oxidation upon light exposure (Additional file 5: Fig. S5). The oxidation of PSI (monitored as ∆ absorbance at 830 nm) is only slightly reduced in the mutant. This result can be explained by a lower photochemical efficiency in zeaxanthin-binding PSI due to the presence of some constitutive zeaxanthin-dependent energy quenching mechanisms, as previously reported in the case of zeaxanthin-binding PSI of *Chlorella vulgaris* [38]. Nevertheless, we can conclude that the effect of the changes of xanthophylls in *Δzl* mutant is more evident in the case of PSII than PSI. The PSI can bind in its antenna complexes also the β carotene while PSII antenna bind only xanthophylls. The pigments distribution in the isolated complexes (Fig. 1c) showed that β carotene was present in similar percentage in Wt and the *Δzl* mutant PSI (b5); this can explain the higher stability of PSI with respect to PSII.

### Photosynthetic activity decreased significantly in the Δzl mutant

In addition to decreased Fv/Fm, the *Δzl* mutant was characterized by lower operating efficiency, ETR, 1−qL, and O_2_ evolution. This indicates that a diminished light-harvesting efficiency results in decreased photosynthetic activity, especially lower light intensities (50 and 100 µmol photons m^−2^ s^−1^), which cannot supply sufficient light energy to the electron transport chain. This may be why the *Δzl* mutant, which has a reduced ability to harvest available photons, initially grows slower than Wt at the lower light regimes.

This O_2_ evolution was different from the results of other mutants with truncated antenna size or with decreased pigment content, where the Pmax was usually reported higher than Wt [6]. The reduced Pmax observed in *Δzl* mutant can be explained considering that the constitutive accumulation of zeaxanthin in *C. reinhardtii* has been previously demonstrated to induce a constitutive quenching mechanism that decreases the photosynthetic efficiency [39]. However, it is important to note that even in the case of *npq2lor1* mutant increased Pmax was measured compared to the Wt [16]: another possible explanation is related to the cell wall. The *npq2lor1* mutant has been derived from strains with an intact cell wall [16]. In contrast, our *Δzl* mutant was obtained from a cell wall-less strain (CC4349) [27]. The periplasmic region situated between the cell wall and cell membrane may play a significant role in the conversion of CO_2_ to bicarbonate by carbonic anhydrase 1, which is present in the periplasmic space [40]. This difference in the carbon uptake and utilization process might be responsible for the observed variations. The other possible explanation is the effect of unexpected gene mutations in the *npq2lor1* mutant, which was isolated as a spontaneous mutation. However, our results cannot be clearly interpreted, and thus a comparative study between the *Δzl* and *npq2lor1* mutants should be performed to clarify this issue.

### CEF compensated for the impaired PSII in the Δzl mutant

The light energy absorbed by the photosystem is directed toward the electron transport chain to generate a proton gradient for ATPase activity and NADPH [31]. The generation of a proton gradient is related to the efficiency of light capture by the photosystems [31] and can be measured by monitoring the carotenoid change in absorption after illumination (ECS). Consistent with decreased photosynthetic efficiency in the *Δzl* mutant, the ECS of the *Δzl* mutant was lower than that of Wt. The CEF contributed less than 10% of the total ECS in the Wt, but approximately 50% in the *Δzl* mutant, suggesting the importance of CEF in generating PMF in the *Δzl* mutant. The ECS signal showed that the *Δzl* mutant generates a lower amount of protons when illuminated consistent with reduced photosynthetic efficiency. When the measure was repeated adding DCMU, to inhibit linear electron transport from PSII to PSI, the proton generation resulted higher in the mutant than in the Wt implying a higher cyclic electron transport (since the linear transport was inhibited). The mutant compensates for the reduced electron flow from the PSII, which in the *Δzl* has a reduced efficiency and stability, increasing the one around PSI to generate enough proton gradient to sustain ATPase activity. Therefore, the *Δzl* mutant seems to compensate for the diminished efficiency and stability of PSII by increasing the electron flow around PSI to generate enough proton gradient to sustain ATPase activity.

### Δzl mutant exhibited an impaired NPQ mechanism

The *Δzl* mutant exhibited a defect in NPQ, a pivotal protective mechanism against light stress, independent of the amount of available inorganic carbon (Fig. 4). This seems to be an effect of both the absence of lutein and the knockout of *ZEP*. Lutein is directly involved in the quenching of chlorophyll-excited states in the antenna via energy transfer from chlorophyll to the S1 state of carotenoids [4, 41]. The *npq1* mutant, which does not accumulate zeaxanthin, showed NPQ similar to that of the Wt, but the double mutant *npq1lor1* showed a considerable decrease in NPQ [42]. According to single-molecule analysis using the 2D-fluorescence correlation analysis, *C. reinhardtii* appears to have pH-dependent and zeaxanthin-dependent quenching systems [39]. Zeaxanthin-dependent quenching was also observed at neutral pH, and a decrease in fluorescence lifetime was observed in the *zep* mutant [39]. This could explain also the strong decrease of Fv/Fm in the *Δzl* mutant.

In addition to the specific roles of lutein and zeaxanthin, changes in xanthophyll content can indirectly affect NPQ by disturbing the assembly and structure of the PSII antenna. Indeed, CP26 knockout mutants defective in antenna structures show a strong decrease in NPQ [43, 44]. The lower PSII antenna content in the *Δzl* mutant could affect the NPQ, because the antenna proteins are the subunits interacting with LHCSR.

### Δzl mutant has an advantage in high-density cultivation at HL conditions

Although a defective PSII light-harvesting complex and an impaired NPQ mechanism, the *Δzl* mutant reached higher cellular concentration than the Wt at 500 μmol photons m^−2^ s^−1^. This is probably related to the 60% lower Chl content in the mutant than its parental strain. The lower chlorophyll content and smaller PSII antenna size in the *Δzl* mutant absorbed less light per cell, allowing better light penetration and greater light availability for the inner layers.

Despite a severe decrease in NPQ in the *Δzl* mutant, the growth was not inhibited at 500 μmol photons m^−2^ s^−1^. The NPQ mechanism is important to protect from changes in light intensity that temporarily saturate photochemistry, whereas it is less important in continuous high-light conditions [45, 46]. In addition, the deficit in NPQ could be compensated for by the antioxidant characteristics of zeaxanthin [47]. Our results showed that the *Δzl* mutant accumulated 60 and 72 times higher zeaxanthin content than Wt in the cells adapted to 100 and 500 μmol photons m^−2^ s^−1^, respectively (Additional file 6: Fig. S6). Zeaxanthin has higher antioxidant activity than other xanthophylls and its constitutive accumulation seems to protect the mutant cells from ROS [48–50]. Indeed, *C. vulgaris* mutants selected for their higher resistance to ROS or accumulation of more carotenoids showed higher productivity in laboratory-scale photobioreactors [9]. In addition, a strain of *C. reinhardtii* engineered to accumulate the strong antioxidant ketocarotenoid astaxanthin as a major carotenoid outcompeted its parental background in terms of biomass productivity [12, 51]. More carotenoids per chlorophyll a in the *Δzl* mutant have more probability of scavenging the ROS generated by chlorophylls. Therefore, the constitutive accumulation of zeaxanthin seems to enable growth under HL conditions by forming a continuously quenched state [42]. Taken together, our results demonstrate that the characteristics of the *Δzl* mutant, which had a smaller antenna size and accumulated large amounts of the antioxidant zeaxanthin, are advantageous in high-density cultivation.

## Materials and methods

### Cell lines and culture conditions

*C. reinhardtii* CC4349 (cw15, mt-) (Wt) and the knockout mutant of zeaxanthin epoxidase and lycopene ε-cyclase (*Δzl2* in the previous study; *Δzl*) [27] were cultivated photoautotrophically. For growth tests at ambient air conditions (0.04% CO_2_), cells were inoculated into 50 mL of high-salt (HS) medium in a 250 mL flask covered with aluminum foil. The carbon source was dissolved naturally in air with agitation. For growth tests under high CO_2_ conditions, cells were inoculated into 80 mL of HS medium in a 250 mL flask closed with a vent plug. The 5% CO_2_ was supplied by bubbling at a rate of 80 mL min^−1^. Due to the rapid evaporation of the medium by bubbling, we increased the culture volume to 80 mL, as opposed to air conditions, using 50 mL of the culture medium. The culture flasks were continuously agitated on an orbital shaker (120 rpm) at 25 ± 1 °C under continuous light conditions (50 ± 5, 100 ± 10, 250 ± 30, and 500 ± 50 μmol photons m^−2^ s^−1^). All experiments were conducted using seed cultures adapted to each light intensity for more than 2 weeks.

### Pigment analysis

The cell culture (0.5 mL) was harvested by centrifugation (20,000 × *g* for 2 min) and the supernatant was discarded. For spectral analysis, the pigments were extracted in 80% (*w*/*w*) acetone. The mixture was centrifuged (20,000 × *g* for 5 min) and the absorbance of the supernatant was determined using a spectrophotometer. The total chlorophyll and carotenoid contents were calculated according to Lichtenthaler’s formula [52]. For HPLC analysis, the pigments were extracted in 0.5 mL of 90% (*w*/*w*) acetone incubation for 1 min. After centrifugation at 20,000 × *g* for 5 min, the supernatant was filtered through a 0.2 μm nylon filter. The filtrate was analyzed using a Shimadzu Prominence high-performance liquid chromatography system (model LC-20AD; Shimadzu, Kyoto, Japan) equipped with a Waters Spherisorb S5 ODS1 cartridge column (4.6 × 250 mm; Waters, Milford, MA, USA) [53]. The pigment concentrations in the extracts were normalized to the sample biomass.

The biomass (dry cell weight) was measured as follows; 10 mL of cell culture was harvested by centrifugation at 2500 × *g* for 10 min at 20 °C. The cells were resuspended and transferred to a pre-weighed 1.5 mL EP tube (A). After lyophilization for at least 2 h until the weight remained unchanged, the tubes were reweighed (B). Net dry weight was calculated as “(B) − (A)”.

### Thylakoid membrane isolation, gel electrophoresis, and immunoblotting

Thylakoid membranes were isolated as previously described [54]: For fractionation of pigment–protein complexes, membranes corresponding to 500 μg of Chlorophylls were washed with 5 mM EDTA and then solubilized in 1 ml of 0.8% α-DM and 10 mM HEPES, pH 7.8. Solubilized samples were then fractionated by ultracentrifugation in a 0.1–1 M sucrose gradient containing 0.03% α-DM and 10 mM HEPES, pH 7.8 (22 h at 280,000 g, 4 °C). SDS–PAGE was performed using the Tris-Tricine buffer system [55] followed by Coomassie blue staining. For immunotitration, thylakoid samples were loaded and electroblotted onto nitrocellulose membranes, which were quantified using an alkaline phosphatase-conjugated antibody system. α-PsaA (AS06 172), α-CP43 (AS11 1787), α-CP26 (AS09 407), and α-LHCII (AS01 003) antibodies were purchased from Agrisera (Sweden). Non-denaturing Deriphat–PAGE was performed as described previously [56]. Thylakoids concentrated at 1 mg mL^−1^ of total chlorophyll were solubilized with a final 0.8% α-DM, and a sample equivalent to 30 μg of total chlorophyll was loaded into each lane.

### Photosynthetic activity

The photosynthetic parameters ΦPSII, ETR, qL, and NPQ were obtained by measuring chlorophyll fluorescence with a DUAL–PAM-100 fluorimeter (Heinz–Walz) at 25 °C in a 1 × 1 cm cuvette mixed by magnetic stirring. ΦPSII, ETR, and qL were measured and calculated according to [30] and [57] at steady-state photosynthesis upon 20 min of illumination. NPQ measurements were performed on dark-adapted intact cells as described previously [43] with the following modifications: cells were adapted to 500 μmol photons m^−2^ s^−1^ only for two days before NPQ measurements, and far-red light exposure was performed for 15 min before turning on the actinic light. PSII functional antenna size was measured from fast chlorophyll induction kinetics induced with a red light of 11 μmol photons m^−2^ s^−1^ on dark-adapted cells (~ 2 × 10^6^ cells/mL) incubated with 50 μM DCMU. The reciprocal of time corresponding to two-thirds of the fluorescence rise (τ2/3) was taken as a measure of the PSII functional antenna size [58]. The oxygen evolution activity of the cultures was measured at 25 °C with a Clark-type O_2_ electrode (Hansatech), as described previously [43]. The PMF upon exposure to different light intensities was measured by Electrochromic Shift (ECS) with MultispeQ v2.0 (PhotosynQ) according to Kuhlgert et al. MultispeQ Beta: A tool for large-scale plant phenotyping connected to an open photosynQ network [59]. Maximum P700 activity was measured after a pulse of saturating light in whole cells treated with DCMU (3-[3,4-dichlorophenyl]-1,1-dimethylurea), ascorbate and methylviologen, as described in (Cecchin Plant Cell and Environment 2021).

### Supplementary Information

**Additional file 1: Figure S1.** Native absorption spectra of the different fractions. Absorption spectra (ABS) in the 350-750 nm region are reported as optical density. For each sample the absorption spectrum was normalized to the maximal absorption peak in the 600–740 nm region.

**Additional file 2: Figure S2.** Functional PSII antenna size. The inset indicates the calculated value normalized to Wt. All the experiment was performed in biological replicates (n = 3).

**Additional file 3: Figure S3.** Nonphotochemical quenching (NPQ) at different light intensities measured in WT and Δzl low light acclimated cells. Cells of Wt (black) and Δzl (red) acclimated to low light conditions were illuminated with actinic lights of 150 (a), 300 (b), 600 (c), 1200 (d) μmol photons m in order to obtain NPQ kinetic. Closed symbol refers to cells grown at atmospheric CO2, while open symbols to cells grown at 5% CO2. Error bars are reported as standard deviation (n=4).

**Additional file 4: Figure S4.** Nonphotochemical quenching (NPQ) at different light intensities measured in WT and Δzl high light acclimated cells. Cells of Wt (black) and Δzl (red) acclimated to high light conditions were illuminated with actinic lights of 150 (a), 300 (b), 600 (c), 1200 (d) μmol photons m in order to obtain NPQ kinetic. Closed symbol refers to cells grown at atmospheric CO2, while open symbols to cells grown at 5% CO2. Error bars are reported as standard deviation (n=4).

**Additional file 5: Figure S5.** Kinetics of P700 oxidation upon light exposure. Light-dependent P700 oxidation of isolated Wt and ∆zl PSI in detergent on a chlorophyll basis. P700 activity was measured after a pulse of saturating light in whole cells treated with DCMU (3-[3,4-dichlorophenyl]-1,1-dimethylurea), ascorbate and methylviologen. Delta absorbance of P700 at 830 nm was used as a measurement of the PSI redox state.

**Additional file 6: Figure S6.** Pigment content (mg g DCW−1). Cells were cultured at 100 ± 10 µmol photons m−2 s−1 and 500 ± 50 µmol photons m−2 s−1. The pigment contents were analyzed using the samples collected 4 days after inoculation. All the experiment was performed in biological replicates (n = 3). Pigment abbreviations; neoxanthin (Nx), violaxanthin (Vx), antheraxanthin (Ax), lutein (Lut), zeaxanthin (Zx), α-carotene (α-Car), and β-carotene (β-Car). Statistical analysis was performed using Student’s t-test (*p < 0.05).

**Additional file 7: Table S1.** Photosynthesis and respiration rates. The parameters extrapolated from the oxygen–light saturation curves are shown in Figure 2e. All the experiment was performed in biological replicates (n > 3). The Δzl mutant values that are significantly different (Student’s t-test, p < 0.05) from wild-type (Wt) are marked with an asterisk (*).

## Data Availability

All data generated or analyzed during this study are included in this published article and its Additional files.

## Abbreviations

Wt: Wild type
ML: Moderate light
HL: High-light
*Δzl*: Zeaxanthin-accumulating mutant
PSI: Photosystem I
PSII: Photosystem II
ROS: Reactive oxygen species
LHC: Light-harvesting complex
LHCI: Light-harvesting complex I
LHCII: Light-harvesting complex II
LCYB: Lycopene β-cyclase
LCYE: Lycopene ε-cyclase
NPQ: Non-photochemical quenching
DCMU: 3-(3,4-Dichlorophenyl)-1,1-dimethylurea
ΦPSII: PSII operating efficiency
ETR: Electron transport rate
PMF: Proton motive force
ECS: Electrochromic shift
CEF: Cyclic electron flow
