# Supplementary figures and images for: Photoautotrophic cultivation of a Chlamydomonas reinhardtii mutant with zeaxanthin as the sole xanthophyll
Source: Biotechnol Biofuels Bioprod. 2024 Mar 14;17:41. doi: 10.1186/s13068-024-02483-8 (PMC10941483; doi:10.1186/s13068-024-02483-8)

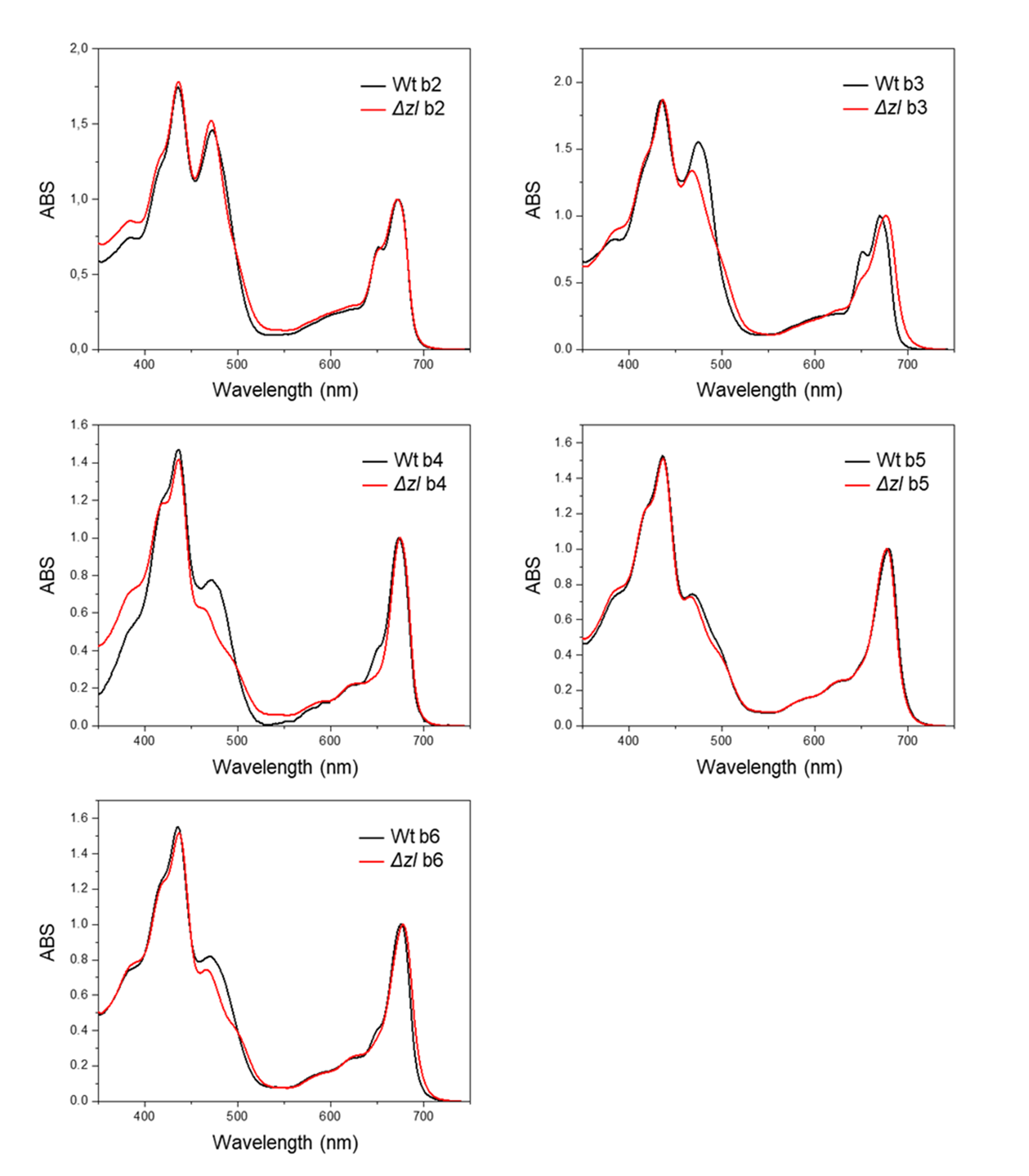

Supplement: Supplementary file 1 — Additional file 1: Figure S1. Native absorption spectra of the different fractions. Absorption spectra (ABS) in the 350-750 nm region are reported as optical density. For each sample the absorption spectrum was normalized to the maximal absorption peak in the 600–740 nm region. [file 13068_2024_2483_MOESM1_ESM.tif]

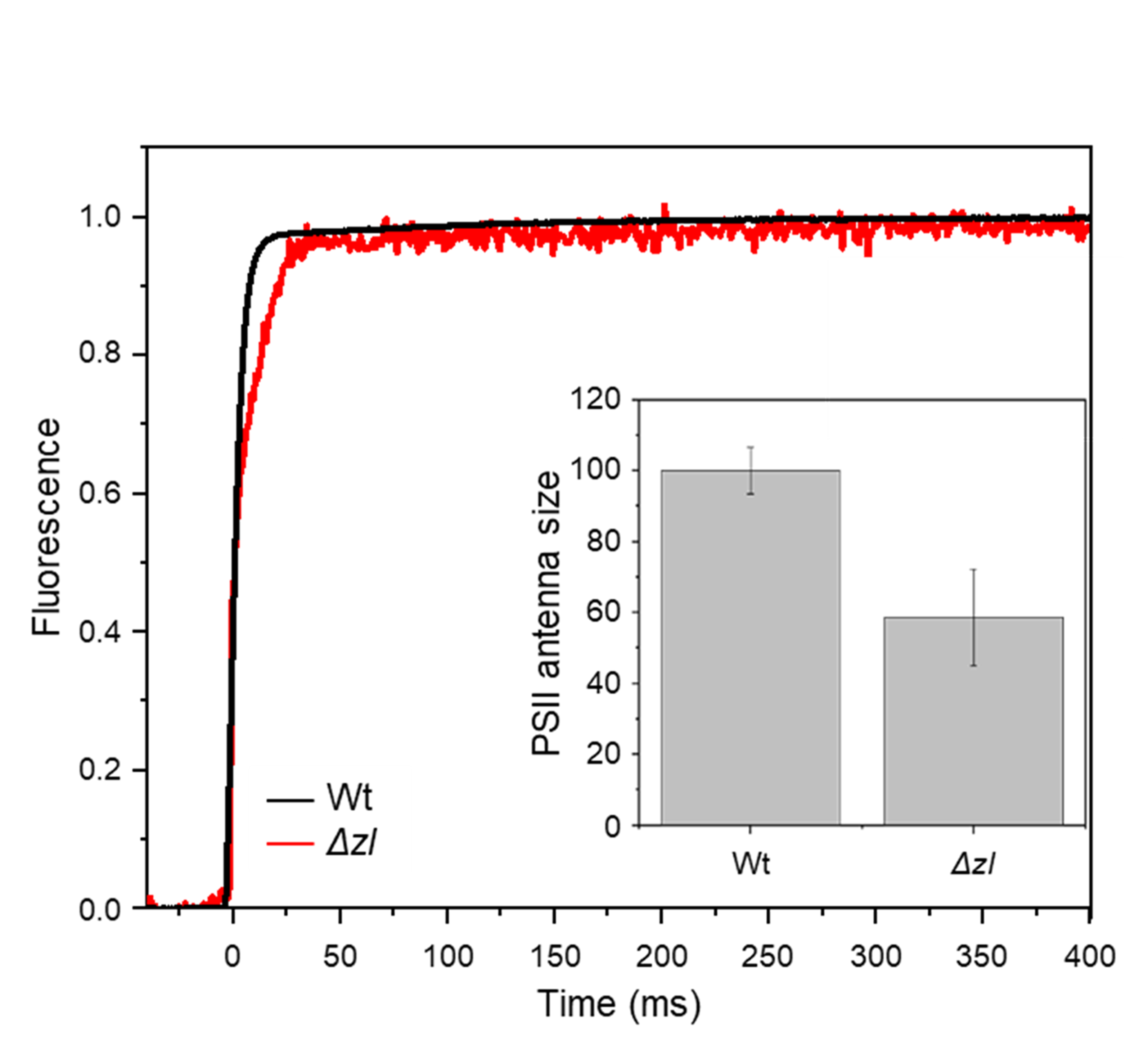

Supplement: Supplementary file 2 — Additional file 2: Figure S2. Functional PSII antenna size. The inset indicates the calculated value normalized to Wt. All the experiment was performed in biological replicates (n = 3). [file 13068_2024_2483_MOESM2_ESM.tif]

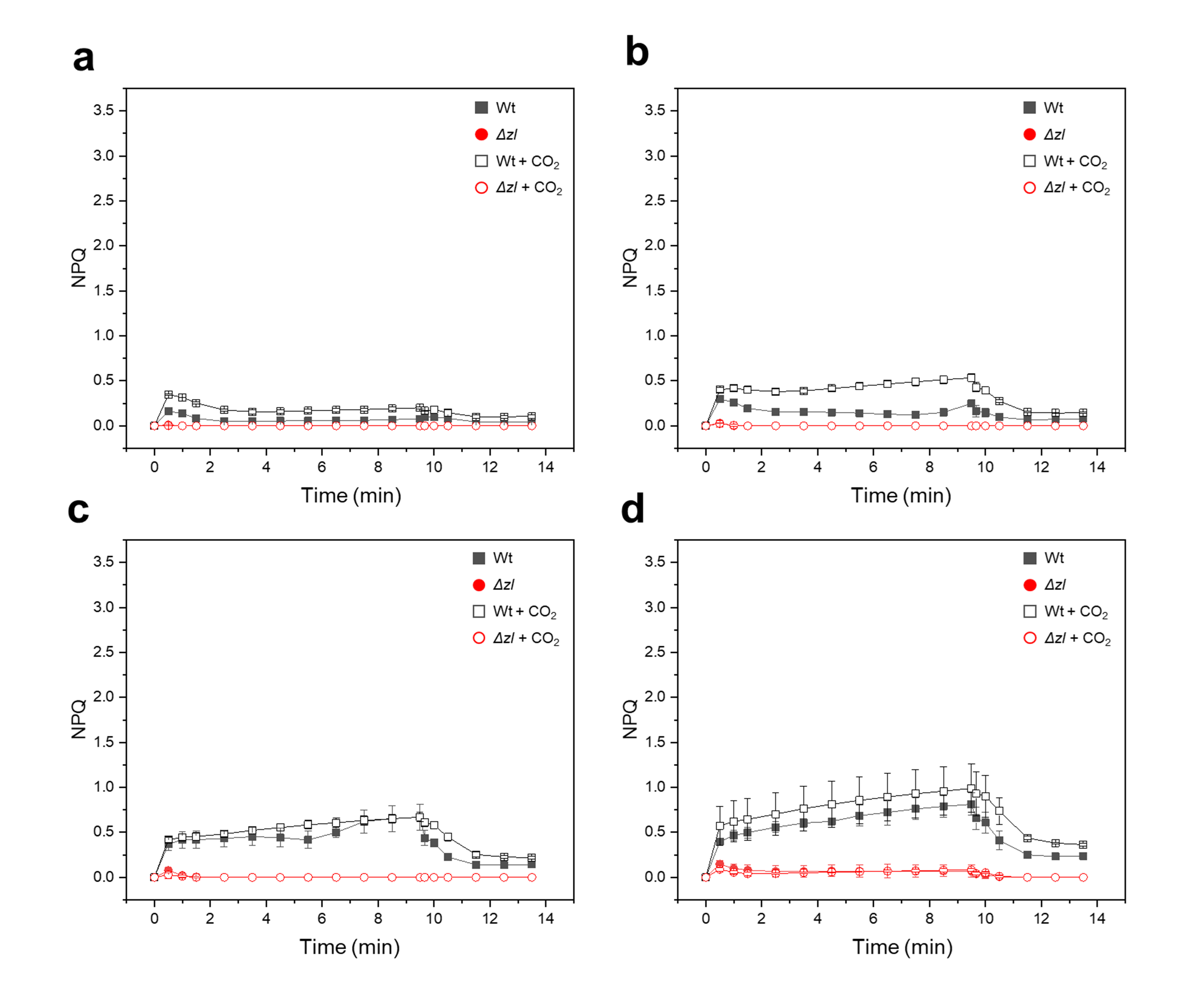

Supplement: Supplementary file 3 — Additional file 3: Figure S3. Nonphotochemical quenching (NPQ) at different light intensities measured in WT and Δzl low light acclimated cells. Cells of Wt (black) and Δzl (red) acclimated to low light conditions were illuminated with actinic lights of 150 (a), 300 (b), 600 (c), 1200 (d) μmol photons m in order to obtain NPQ kinetic. Closed symbol refers to cells grown at atmospheric CO2, while open symbols to cells grown at 5% CO2. Error bars are reported as standard deviation (n=4). [file 13068_2024_2483_MOESM3_ESM.tif]

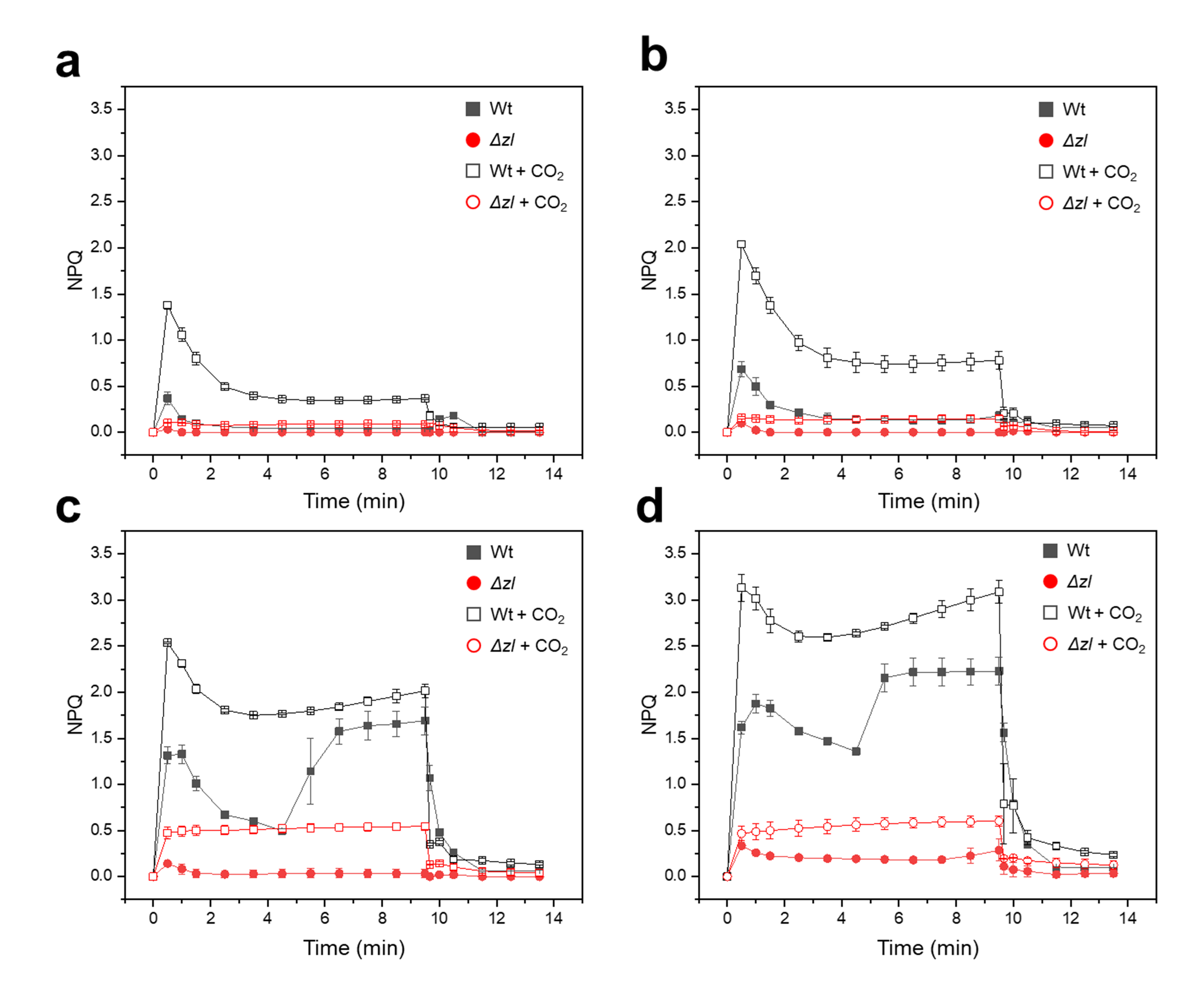

Supplement: Supplementary file 4 — Additional file 4: Figure S4. Nonphotochemical quenching (NPQ) at different light intensities measured in WT and Δzl high light acclimated cells. Cells of Wt (black) and Δzl (red) acclimated to high light conditions were illuminated with actinic lights of 150 (a), 300 (b), 600 (c), 1200 (d) μmol photons m in order to obtain NPQ kinetic. Closed symbol refers to cells grown at atmospheric CO2, while open symbols to cells grown at 5% CO2. Error bars are reported as standard deviation (n=4). [file 13068_2024_2483_MOESM4_ESM.tif]

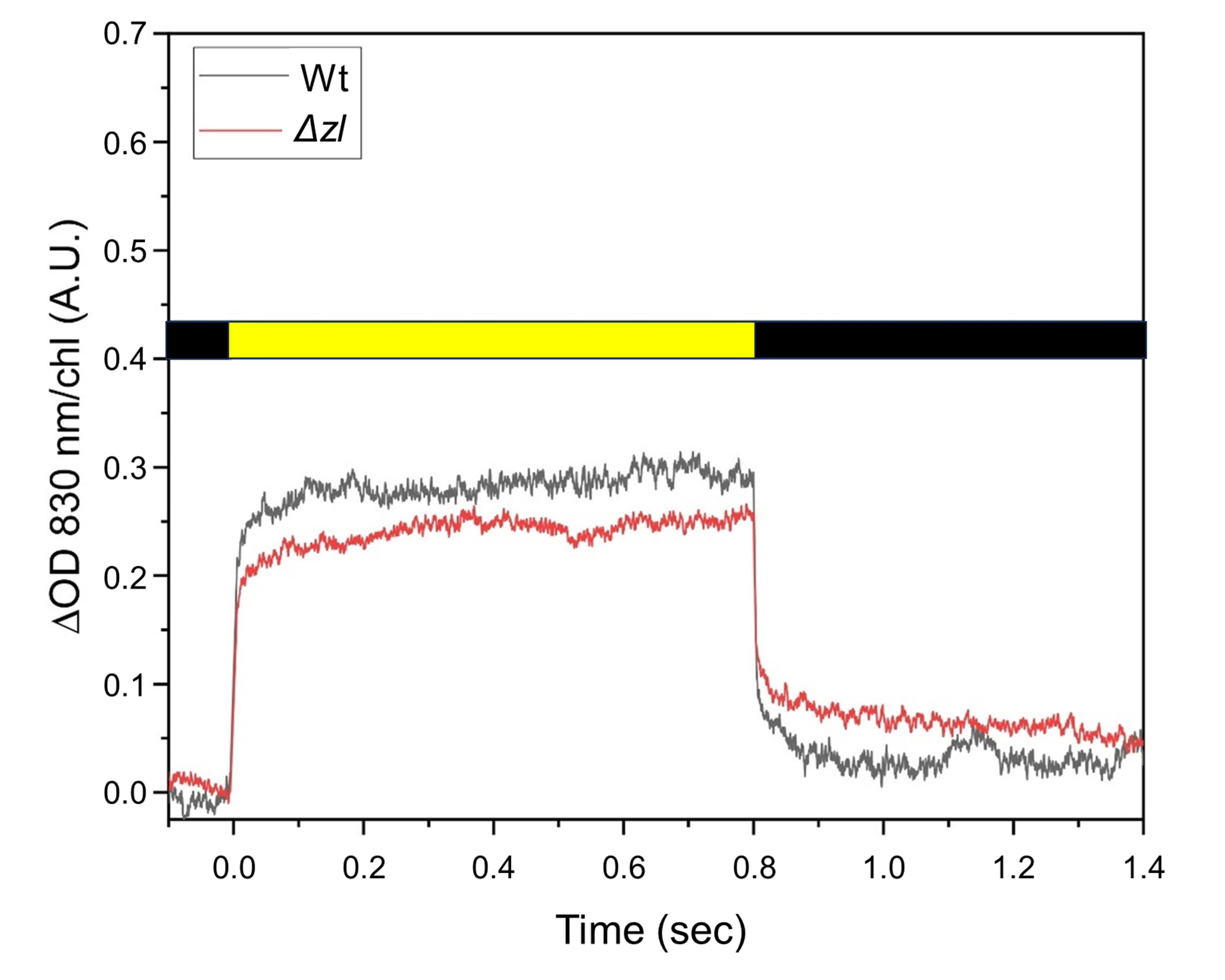

Supplement: Supplementary file 5 — Additional file 5: Figure S5. Kinetics of P700 oxidation upon light exposure. Light-dependent P700 oxidation of isolated Wt and ∆zl PSI in detergent on a chlorophyll basis. P700 activity was measured after a pulse of saturating light in whole cells treated with DCMU (3-[3,4-dichlorophenyl]-1,1-dimethylurea), ascorbate and methylviologen. Delta absorbance of P700 at 830 nm was used as a measurement of the PSI redox state. [file 13068_2024_2483_MOESM5_ESM.tif]

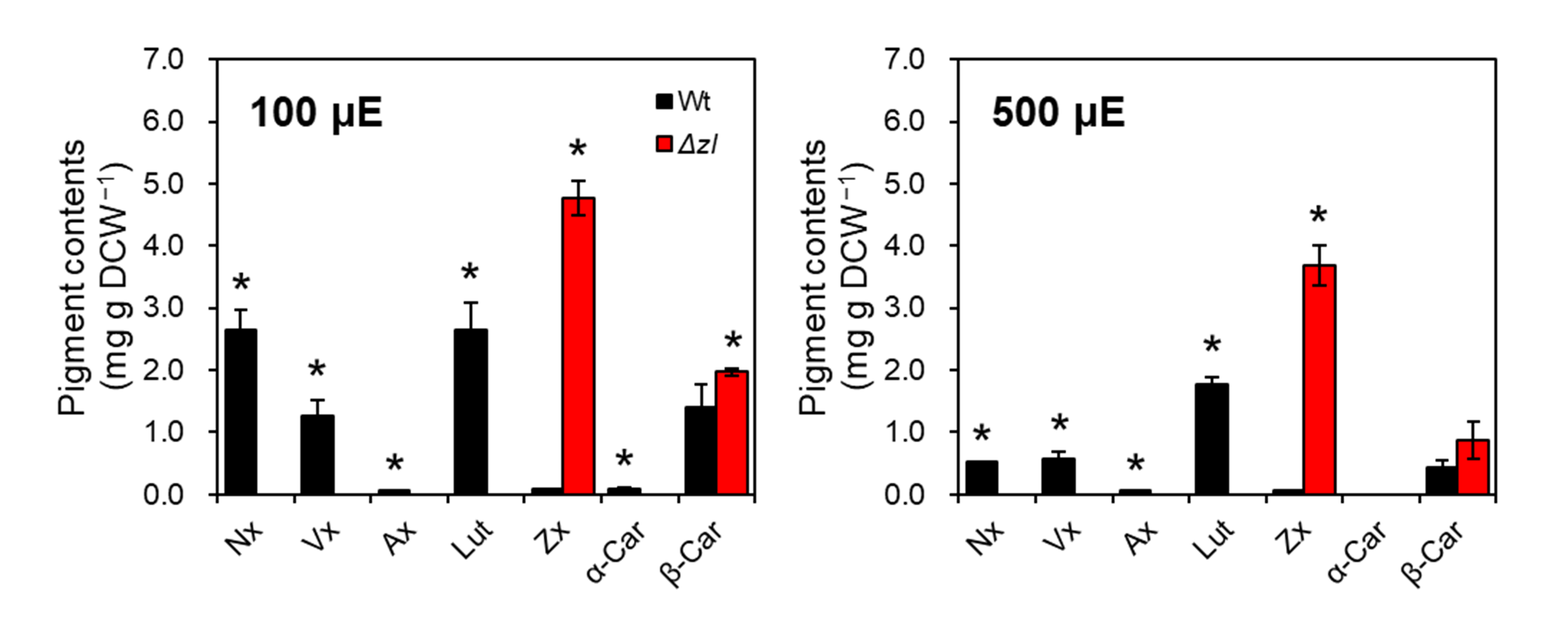

Supplement: Supplementary file 6 — Additional file 6: Figure S6. Pigment content (mg g DCW−1). Cells were cultured at 100 ± 10 µmol photons m−2 s−1 and 500 ± 50 µmol photons m−2 s−1. The pigment contents were analyzed using the samples collected 4 days after inoculation. All the experiment was performed in biological replicates (n = 3). Pigment abbreviations; neoxanthin (Nx), violaxanthin (Vx), antheraxanthin (Ax), lutein (Lut), zeaxanthin (Zx), α-carotene (α-Car), and β-carotene (β-Car). Statistical analysis was performed using Student’s t-test (*p < 0.05). [file 13068_2024_2483_MOESM6_ESM.tif]
