# Supplementary material for: Photoautotrophic cultivation of a Chlamydomonas reinhardtii mutant with zeaxanthin as the sole xanthophyll
Source: Biotechnol Biofuels Bioprod. 2024 Mar 14;17:41. doi: 10.1186/s13068-024-02483-8 (PMC10941483; doi:10.1186/s13068-024-02483-8)
Supplement: Supplementary file 7 — Additional file 7: Table S1. Photosynthesis and respiration rates. The parameters extrapolated from the oxygen–light saturation curves are shown in Figure 2e. All the experiment was performed in biological replicates (n > 3). The Δzl mutant values that are significantly different (Student’s t-test, p < 0.05) from wild-type (Wt) are marked with an asterisk (*). [file 13068_2024_2483_MOESM7_ESM.docx]

**Supplementary Table S1. Photosynthesis and respiration rates.** The parameters extrapolated from the oxygen–light saturation curves are shown in **Figure 2e**. All the experiment was performed in biological replicates (n > 3). The *Δzl* mutant values that are significantly different (Student’s t-test, p < 0.05) from wild-type (Wt) are marked with an asterisk (*).

| Parameters | Wt | *Δzl* |
| --- | --- | --- |
| Respiration in the dark  (O_2_ nmol 10^6^ cell^−2^ min^−1^) | -0.43 ± 0.06 | -0.19 ± 0.01 * |
| Pmax (O_2_ nmol μg Chl^−1^ min^−1^) | 3.34 ± 0.23 | 2.90 ± 0.35 |
| Half-saturation intensity  (μmol photons m^−2^ s^−1^) | 321 ± 7 | 521 ± 17 * |
| Slope of linear increase | 0.0056 ± 0.0001 | 0.0031 ± 0.0002 * |
